# From symptomatic management to precision medicine: A bibliometric analysis and visualization of global research trends in primary hyperoxaluria (1990–2025)

**DOI:** 10.1097/MD.0000000000051250

**Published:** 2026-10-23

**Authors:** Xiao Han, Shaozhong Xian, Yukun Liu

**Affiliations:** a Department of Urology, Beijing Luhe Hospital, Capital Medical University, Beijing, China.

**Keywords:** bibliometric analysis, gene editing, knowledge mapping, precision medicine, primary hyperoxaluria, RNA interference

## Abstract

**Background:**

Primary hyperoxaluria (PH) is a rare, inherited metabolic disorder characterized by excessive oxalate production and progressive renal impairment. This study aimed to systematically analyze global research trends in PH from 1990 to 2025 using bibliometric methods with a focus on the evolution from conventional management strategies to emerging precision medicine approaches.

**Methods:**

Publications related to PH were retrieved from the Web of Science Core Collection (1990–September 2025; retrieval date: September 15, 2025). Bibliometric and visualization analyses were performed using VOSviewer (version 1.6.20), CiteSpace (version 6.3.R1), and R software (version 4.3.2) to assess publication trends, collaborative networks, co-citation patterns, and keyword evolution.

**Results:**

A total of 1630 publications were included in the analysis, demonstrating a marked increase in research output after 2020. The United States contributed the largest proportion of publications and had the highest total citation count, while European countries and China showed increasing research participation. Keyword and co-citation analyses revealed an early focus on clinical manifestations and transplantation-based management, followed by a transition toward molecular mechanisms and ribonucleic acid (RNA) interference-based therapies. Recently, RNA interference-based therapies and related metabolic targets have emerged as major research hotspots, whereas gene-editing approaches remain an emerging preclinical direction.

**Conclusion:**

Global research on PH has undergone a clear thematic shift from symptomatic management to molecularly targeted RNA-based therapies. RNA interference represents the current research frontier, while gene-editing technologies are still in the preclinical stage and represent a long-term potential direction for future research. This study provides a comprehensive overview of the intellectual structure and evolving landscape of PH research.

## 1. Introduction

Primary hyperoxaluria (PH) is a rare autosomal recessive metabolic disorder caused by hepatic enzymatic defects that lead to excessive endogenous oxalate production. Clinically, PH commonly manifests as recurrent urolithiasis, nephrocalcinosis, progressive kidney dysfunction, and systemic oxalosis in advanced cases.^[1]^ Although PH is uncommon, its clinical burden is substantial because delayed diagnosis and persistent oxalate accumulation can result in irreversible kidney damage.

Advances in molecular genetics have improved our understanding of the genetic basis and phenotypic heterogeneity of PH. PH is generally classified into 3 major subtypes, according to the underlying pathogenic genes. PH type 1, caused by mutations in alanine–glyoxylate aminotransferase (AGXT), is the most common and severe form. PH types 2 and 3 are associated with mutations in glyoxylate reductase/hydroxypyruvate reductase (GRHPR) and 4-hydroxy-2-oxoglutarate aldolase 1 (HOGA1), respectively.^[2,3]^ Genetic testing has become essential for accurate diagnosis, subtype classification, and individualized management.

Current treatment strategies for PH include supportive management and targeted intervention. Conventional approaches, such as hyperhydration, pyridoxine supplementation, citrate therapy, and urological procedures, may help reduce stone formation and manage complications; however, they do not fully correct hepatic oxalate overproduction.^[4]^ For patients with advanced PH type 1, combined liver–kidney transplantation has historically been used as a disease-modifying treatment by replacing the defective hepatic enzyme source; however, this approach is invasive and associated with substantial clinical burden.^[5]^

In recent years, ribonucleic acid (RNA) interference-based therapies have introduced a new therapeutic direction for PH. Agents such as lumasiran and nedosiran target key enzymes involved in hepatic oxalate production and have shown the ability to reduce urinary oxalate levels in patients with PH.^[6–8]^ These developments indicate a shift in PH research and treatment from symptom control and organ replacement to pathway-targeted and precise therapeutic strategies.

Despite the increasing clinical and translational interest in PH, existing reviews have primarily focused on disease mechanisms, diagnosis, and therapeutic advances, whereas the overall evolution of the research landscape has not been systematically evaluated. Bibliometric analysis provides a quantitative approach to evaluating publication trends, collaboration patterns, knowledge structures, and emerging research hotspots,^[9,10]^ offering insights that conventional narrative reviews cannot provide. Therefore, this study conducted a bibliometric analysis and visualization of the PH-related literature published between 1990 and 2025 to provide a comprehensive overview of global research trends and identify current and potential future research directions.

## 2. Materials and methods

### 2.1. Data source and search strategy

Bibliographic data were retrieved from the Web of Science Core Collection and Science Citation Index Expanded on September 15, 2025. A topic search was conducted using the following strategy: TS = (“primary hyperoxaluria” OR “hyperoxalurias, primary” OR “oxalurias, primary” OR “primary oxalosis”). The search period was limited to January 1, 1990, to September 15, 2025. To verify the sensitivity of the primary search strategy, we conducted a supplementary topic search using the following query: TS = ((“AGXT” OR “GRHPR” OR “HOGA1” OR “lumasiran” OR “nedosiran”) AND (“hyperoxaluria” OR “oxalosis”)). This supplementary search retrieved 1336 records. After removing records already identified by the primary search, no additional eligible publications were found. Full records and cited references were exported in a plain text format for subsequent bibliometric analysis.

### 2.2. Inclusion and exclusion criteria

Publications were included if they were related to PH, written in English, and indexed in the Web of Science Core Collection with complete bibliographic information. Eligible publication types were restricted to original articles and reviews. Conference proceedings, meeting abstracts, editorial materials, letters, corrections, news items, duplicate records, and records with incomplete information were excluded. After screening, 1630 publications were included in the final analysis (Fig. 1).

**Figure 1.**
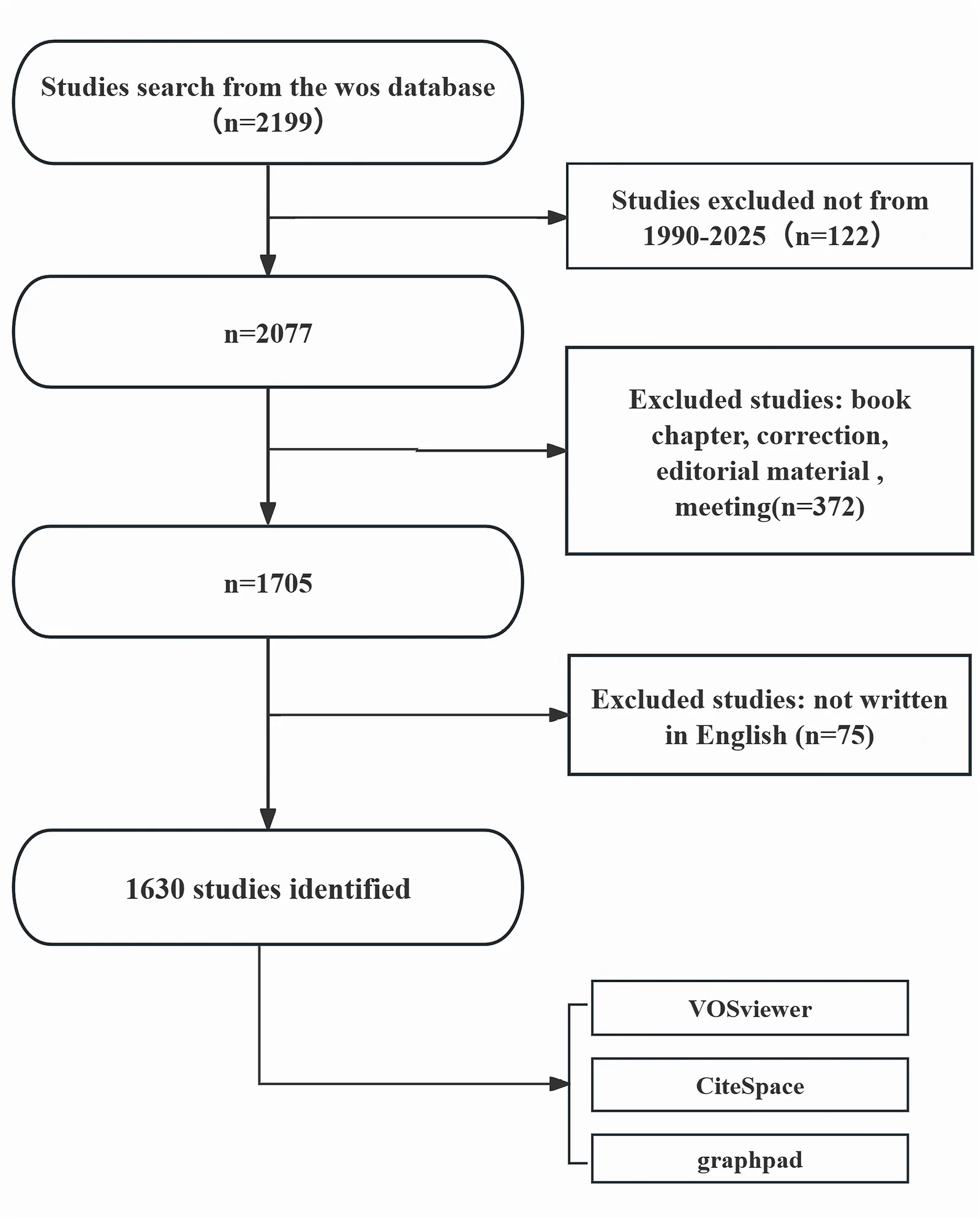
Flow diagram of literature retrieval and screening. The flow diagram shows the process of database retrieval, eligibility screening, exclusion, and final inclusion of publications for bibliometric analysis.

### 2.3. Data preprocessing

Before the analysis, bibliographic data were standardized to improve accuracy. Two independent reviewers screened all records, and discrepancies were resolved through discussion. Data cleaning included unification of author names, institutional affiliations, and keyword harmonization to minimize duplicates and inconsistencies. Keywords with similar meanings were merged where appropriate, such as “AGXT mutation” and “AGXT gene mutation.”

### 2.4. Bibliometric analysis

A descriptive bibliometric analysis was performed using R software (version 4.3.2; R Foundation for Statistical Computing) and the Bibliometrix package. VOSviewer (version 1.6.20; Centre for Science and Technology Studies, Leiden University) was used to construct co-authorship, co-citation, and keyword co-occurrence networks, with the minimum keyword occurrence threshold set at 5.^[11]^ CiteSpace (version 6.3.R1; Drexel University) was used to analyze authors, institutions, references, and keywords separately.^[12]^ The time span was set to 1990 to 2025, with a 1-year time slice. The term source was retained at the default setting, and the node types were selected separately for each analysis. The selection criterion was set to the *g*-index with *k* = 25, while the remaining parameters, including pruning settings, were retained at their default values. Clusters were labeled using the log-likelihood ratio method. Citation and keyword bursts were detected using the default burst-detection parameters, and the top 50 items with the strongest bursts were presented. Institutional name variants were standardized through synonym replacement in CiteSpace and subsequent manual verification. Publication trends were visualized using GraphPad Prism.

### 2.5. Ethical considerations

This study used publicly available bibliographic data and did not involve human participants or animals. Therefore, ethical approval and informed consent were not required.

## 3. Results

### 3.1. Publication trends

A total of 1630 publications related to PH were identified from 1990 to 2025. Annual publication output remained relatively stable before 2008, followed by gradual growth from 2008 to 2020. A marked increase was observed after 2020, with publication output reaching its highest level by 2023. This trend suggests increasing academic interest in PH, particularly after the clinical translation of RNA interference-based therapies (Fig. 2A).

**Figure 2.**
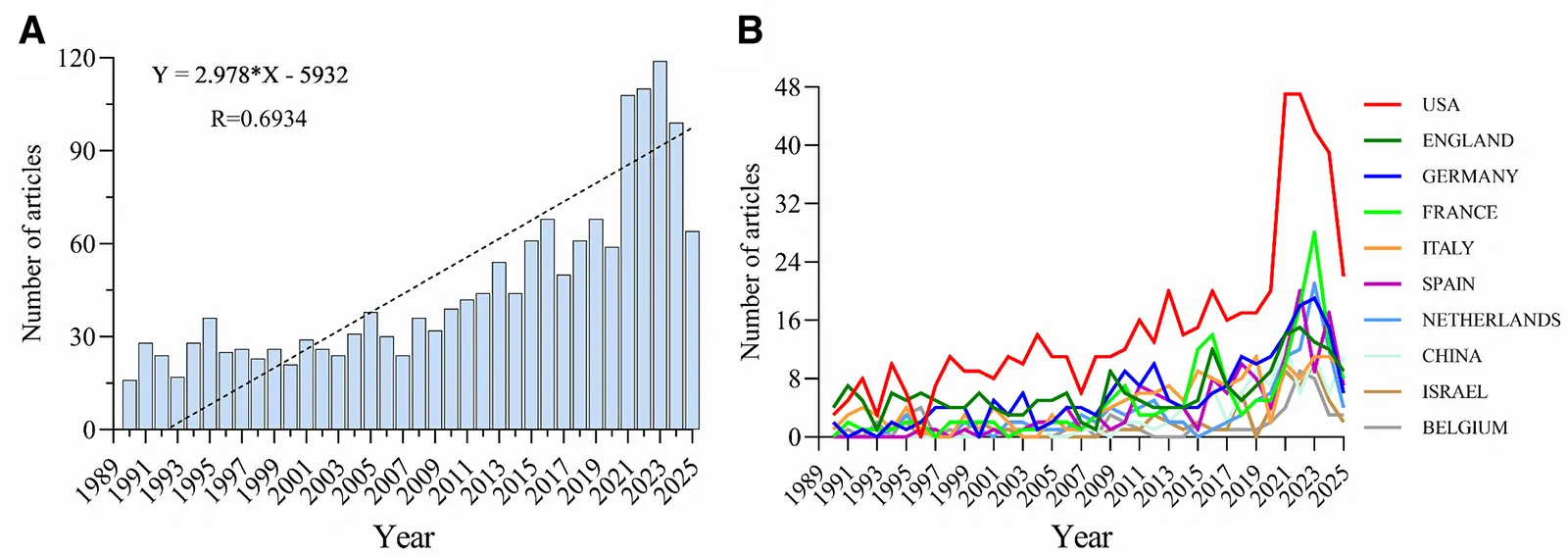
Publication trends and geographic distribution of primary hyperoxaluria research from 1990 to 2025. (A) Annual number of publications. (B) Publication output of the top 10 most productive countries/regions. The 2025 publication count represents partial-year data collected through September 15, 2025.

### 3.2. Country and institutional contributions

Eighty-four countries contributed to PH research. The United States ranked first in both publication output and total citation count, accounting for approximately one-third of all publications. European countries, including England, Germany, France, Italy, Spain, and the Netherlands, also showed strong research productivity (Table 1 and Fig. 2B). China showed increasing publication output, although its total citation count remained lower than that of the established research hubs (Table S1, Supplemental Digital Content 1).

**Table 1:** Top 10 most productive countries/regions in primary hyperoxaluria research.

Rank	Country/region	Article counts	Centrality	Percentage (%)	Citations	Citation per publication
1	United States	542	0.09	33.25	15,188	28.02
2	England	222	0.15	13.62	7201	32.44
3	Germany	213	0.25	13.07	5988	28.11
4	France	176	0.49	10.80	4962	28.19
5	Italy	162	0.08	9.94	3488	21.53
6	Spain	143	0.06	8.77	2640	18.46
7	Netherlands	118	0.04	7.24	3440	29.15
8	China	99	0	6.07	936	9.45
9	Israel	67	0.19	4.11	1726	25.76
10	Belgium	60	0.2	3.68	1488	24.80

Collaboration network analysis showed strong cooperation between North America and Europe, whereas inter-regional collaboration involving Asia remained relatively limited (Fig. 3A). At the institutional level, the Mayo Clinic was the most productive institution, followed by the University of London, University College London, and Universidad de La Laguna (Table 2 and Fig. 3B).

**Table 2:** Top 10 most productive institutions in primary hyperoxaluria research.

Rank	Institution	Country	Number of studies	Total citations	Average citations
1	Mayo Clinic	USA	136	3807	27.99
2	University of London	England	130	1696	13.05
3	University College London	England	122	2291	18.78
4	Universidad de La Laguna	Spain	90	1937	21.52
5	University of Amsterdam	Netherlands	80	2070	25.88
6	Centro de Investigacion Biomedica en Red (CIBER)	Spain	73	1485	20.34
7	Assistance Publique Hopitaux Paris	France	66	1098	16.64
8	University Hospital of the Canary Islands	Spain	66	980	14.85
9	Emma Children’s Hospital	Netherlands	61	148	2.43
10	Institut National de la Sante et de la Recherche Medicale	France	61	1037	17.00

**Figure 3.**
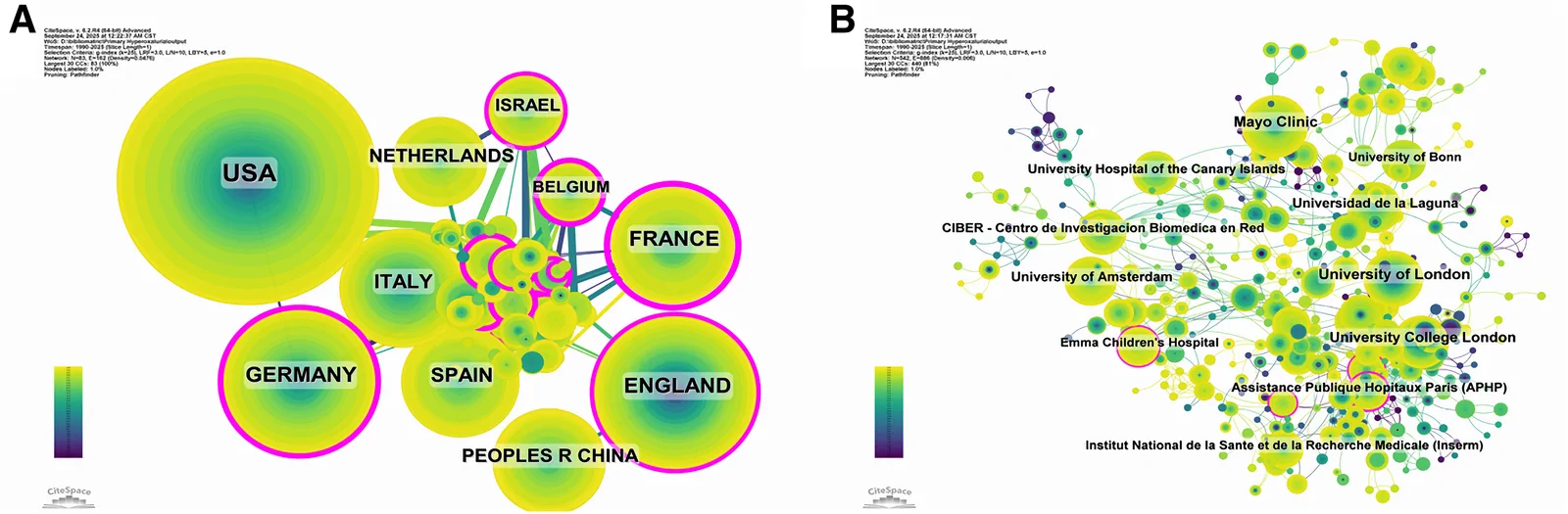
Collaboration networks in primary hyperoxaluria research. (A) Country/region collaboration network. (B) Institutional collaboration network. Node size represents publication output, and links indicate collaborative relationships.

### 3.3. Journal and co-citation analysis

The PH-related studies were published in 502 journals. *Pediatric Nephrology* was the most productive journal, followed by *Nephrology Dialysis Transplantation* and the *Journal of the American Society of Nephrology* (Tables 3 and S2, Supplemental Digital Content 2). Journal co-citation analysis further identified the most influential journals in the field (Tables 4 and S2, Supplemental Digital Content 2).

**Table 3:** Top 10 journals publishing primary hyperoxaluria-related studies.

Rank	Journal	Article counts	Percentage (n= = 1630)	IF	Quartile in category
1	*Pediatric Nephrology*	171	10.49	2.6	Q1
2	*Nephrology Dialysis Transplantation*	77	4.72	5.6	Q1
3	*Journal of the American Society of Nephrology*	66	4.05	9.4	Q1
4	*Kidney International*	48	2.94	12.6	Q1
5	*Pediatric Transplantation*	44	2.70	1.4	Q3
6	*American Journal of Kidney Diseases*	40	2.45	8.2	Q1
7	*Journal of Urology*	29	1.78	6.8	Q1
8	*Transplantation*	28	1.72	5.0	Q1
9	*American Journal of Transplantation*	27	1.66	8.2	Q1
10	*Urolithiasis*	25	1.53	2.2	Q2

**Table 4:** Top 10 co-cited journals in primary hyperoxaluria research.

Rank	Cited journal	Co-citation frequency (n)	Impact factor (2024)	Quartile in category
1	*Kidney International*	797	12.6	Q1
2	*Nephrology Dialysis Transplantation*	683	5.6	Q1
3	*The New England Journal of Medicine*	643	78.5	Q1
4	*Pediatric Nephrology*	562	2.6	Q1
5	*Journal of the American Society of Nephrology*	544	9.4	Q1
6	*American Journal of Kidney Diseases*	442	8.2	Q1
7	*Clinical Journal of the American Society of Nephrology*	378	7.1	Q1
8	*Proceedings of the National Academy of Sciences of the United States of America*	366	9.1	Q1
9	*Journal of Biological Chemistry*	361	3.9	Q2
10	*American Journal of Transplantation*	359	8.2	Q1

Co-citation analysis showed that *Kidney International*, *Nephrology Dialysis Transplantation*, and *The New England Journal of Medicine* were the most influential journals. These journals represent the core intellectual base of PH research, covering clinical nephrology, transplantation, and emerging therapeutic advances (Table 4, Fig. 4, Table S2, Supplemental Digital Content 2). Dual-map overlay analysis further illustrated that the primary citation trajectories originate from journals specializing in molecular biology and health sciences, gravitating toward medicine and clinical research disciplines (Fig. 5).

**Figure 4.**
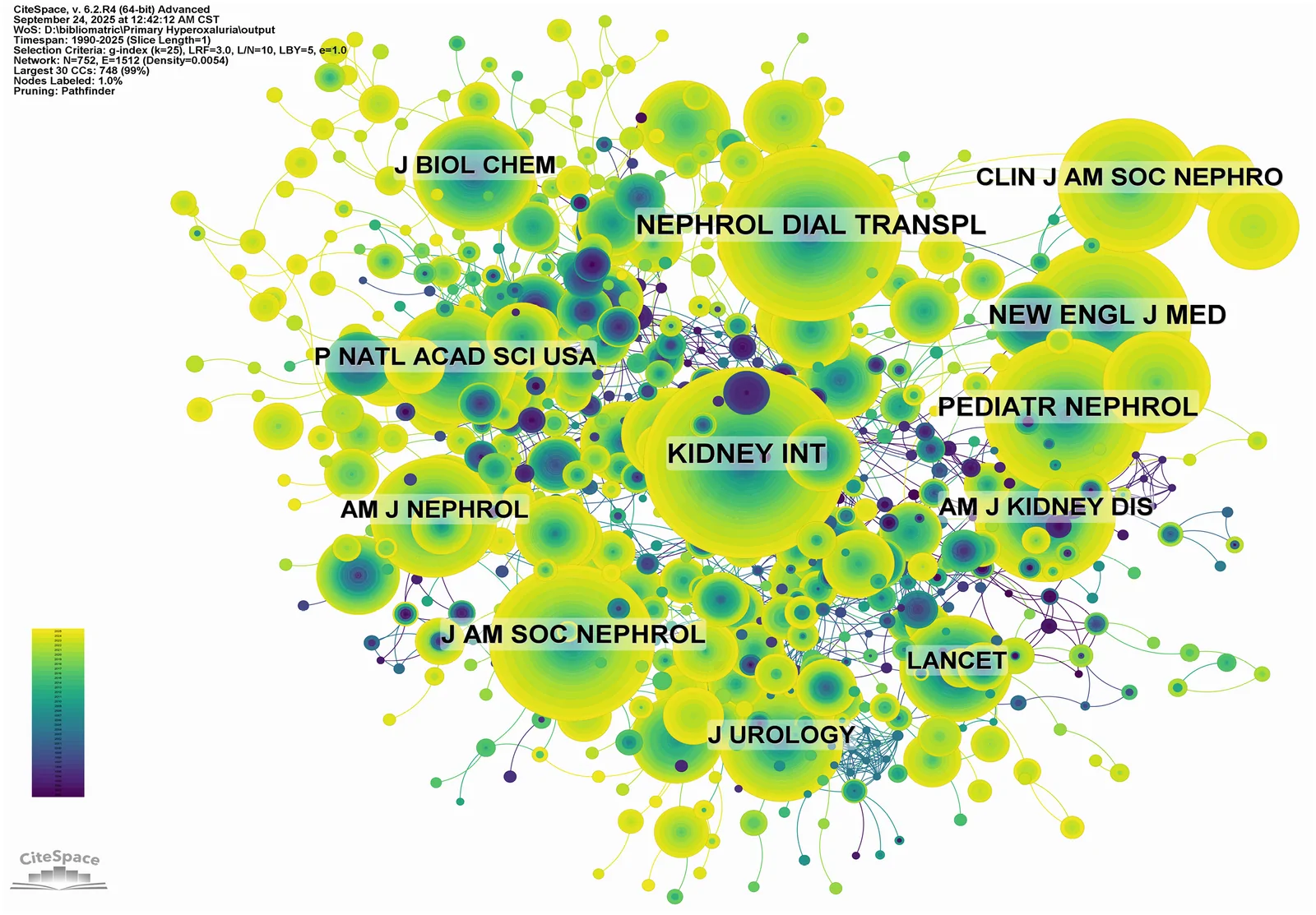
Co-citation network of journals in primary hyperoxaluria research. Node size represents co-citation frequency, and links indicate co-citation relationships among journals.

**Figure 5.**
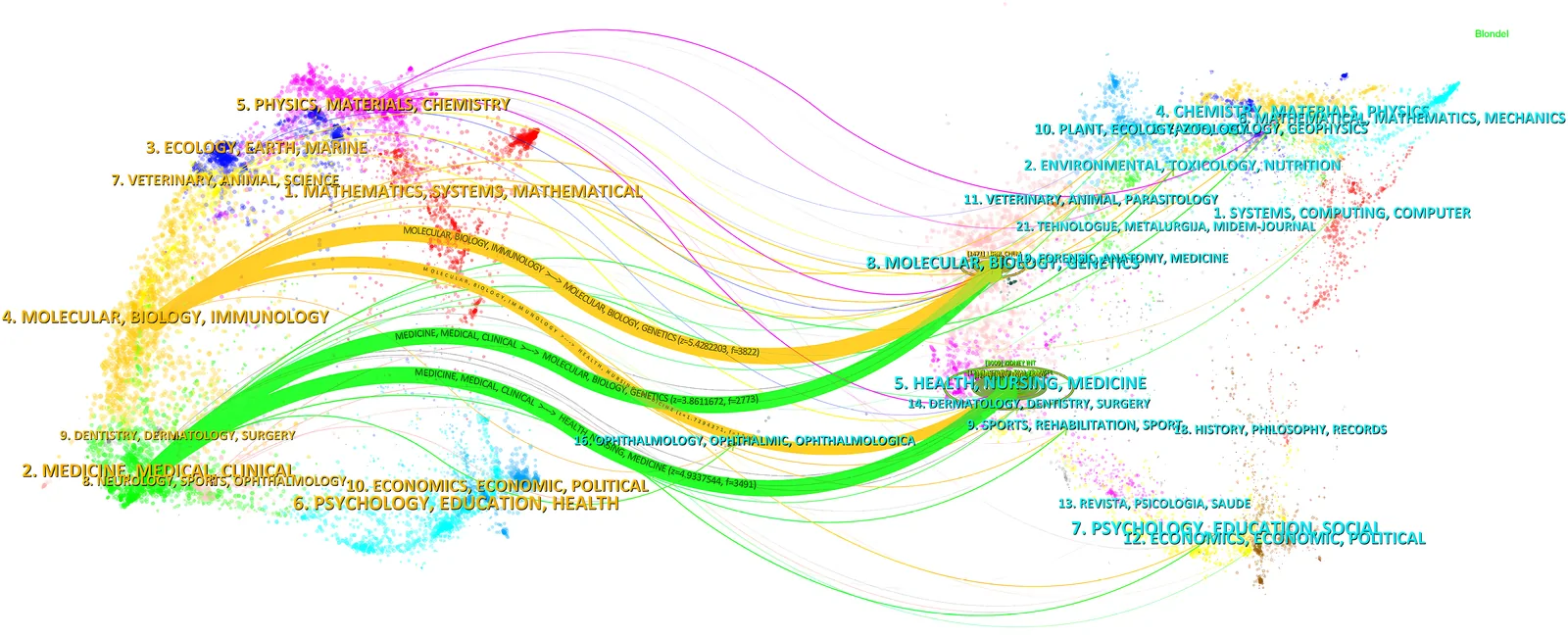
Dual-map overlay of journals in primary hyperoxaluria research. The map illustrates citation pathways between citing journals and cited journals, reflecting the disciplinary distribution of primary hyperoxaluria research.

### 3.4. Author and reference analysis

Hoppe B, Lieske JC, and Salido E were the most productive authors, while author co-citation analysis identified Cochat P, Danpure CJ, and Hoppe B as highly influential contributors to the field (Table 5, Fig. 6, Table S3, Supplemental Digital Content 3). Among the co-cited references, the 2021 *New England Journal of Medicine* study on lumasiran was the most frequently co-cited reference (Table 6) and exhibited the strongest citation burst (strength = 47.58) from 2022 to 2025 (Fig. 7), highlighting its substantial recent influence on PH research. Together with the earlier clinical guidance by Cochat et al, this study represents an important intellectual foundation for modern PH management.^[13–15]^

**Table 5:** Top 10 productive authors and co-cited authors in primary hyperoxaluria research.

Rank	Author	Count	Rank	Co-cited author	Co-citation frequency (n)
1	Hoppe B	90	1	Cochat P	558
2	Lieske JC	66	2	Danpure CJ	503
3	Salido E	51	3	Hoppe B	466
4	Milliner DS	47	4	Monico CG	248
5	Bacchetta J	45	5	Milliner DS	235
6	Cellini B	45	6	Purdue PE	205
7	Cochat P	45	7	Harambat J	196
8	Frishberg Y	44	8	Watts RW	177
9	Danpure CJ	42	9	Hopp K	172
10	Groothoff J	37	10	Van W	168

**Table 6:** Top 10 most frequently co-cited references in primary hyperoxaluria research.

Rank	Title	Journal	Co-citation frequency (n)	
1	Lumasiran, an RNAi Therapeutic for Primary Hyperoxaluria Type 1	*The New England Journal of Medicine*		123
2	Primary hyperoxaluria Type 1: indications for screening and guidance for diagnosis and treatment	*Nephrology Dialysis Transplantation*		61
3	Primary hyperoxaluria	*The New England Journal of Medicine*		57
4	Clinical practice recommendations for primary hyperoxaluria: an expert consensus statement from ERKNet and OxalEurope	*Nature Reviews Nephrology*		56
5	An Investigational RNAi Therapeutic Targeting Glycolate Oxidase Reduces Oxalate Production in Models of Primary Hyperoxaluria	*Journal of the American Society of Nephrology*		51
6	Specific Inhibition of Hepatic Lactate Dehydrogenase Reduces Oxalate Production in Mouse Models of Primary Hyperoxaluria	*Molecular Therapy*		48
7	Patients with primary hyperoxaluria type 2 have significant morbidity and require careful follow-up	*Kidney International*		48
8	Safety, pharmacodynamics, and exposure-response modeling results from a first-in-human phase 1 study of nedosiran (PHYOX1) in primary hyperoxaluria	*Kidney International*		46
9	Phenotype-Genotype Correlations and Estimated Carrier Frequencies of Primary Hyperoxaluria	*Journal of the American Society of Nephrology*		46
10	End Points for Clinical Trials in Primary Hyperoxaluria	*Clinical Journal of the American Society of Nephrology*		46

**Figure 6.**
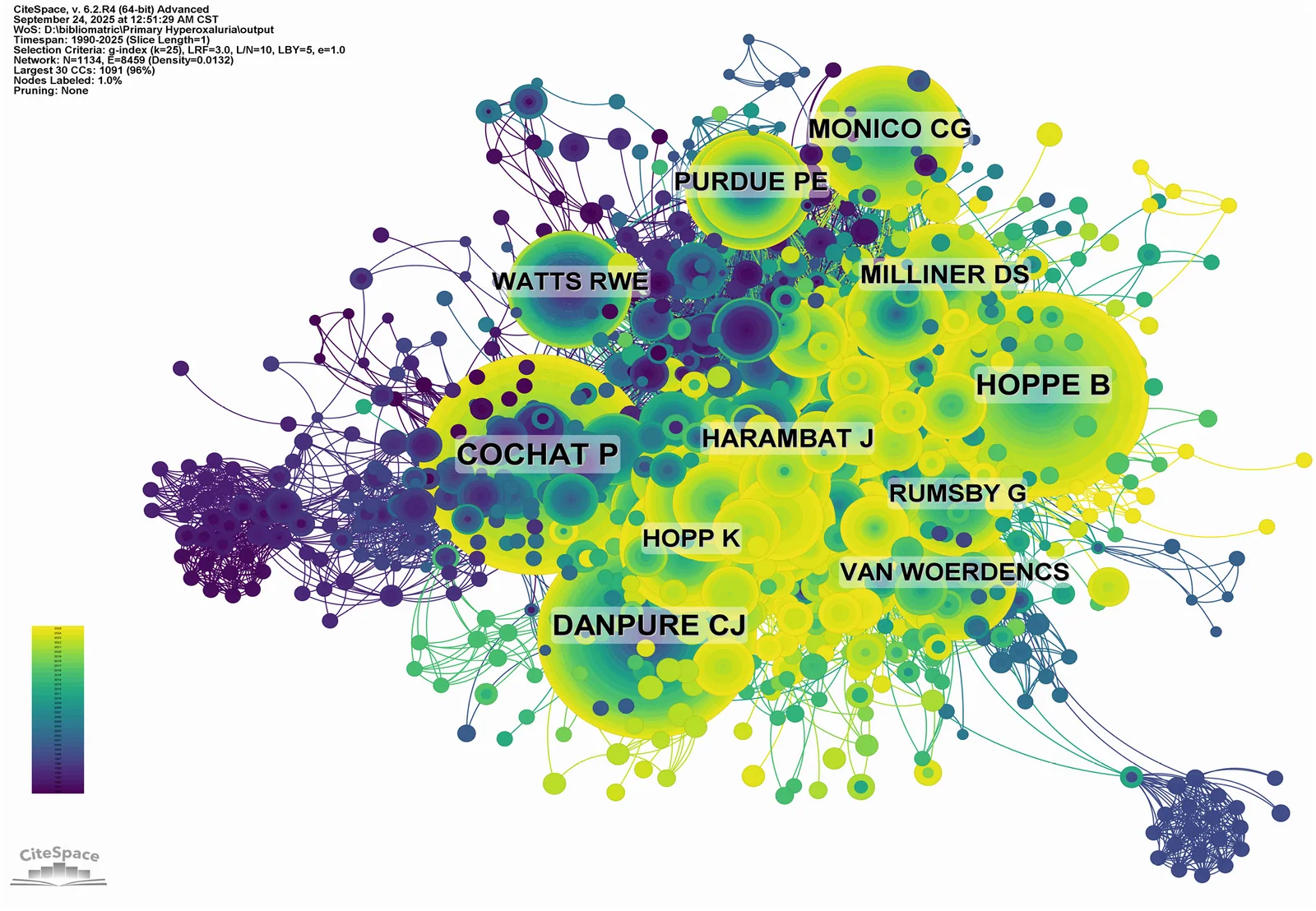
Co-citation network of authors in primary hyperoxaluria research. Node size represents co-citation frequency, and links indicate co-citation relationships among authors.

**Figure 7.**
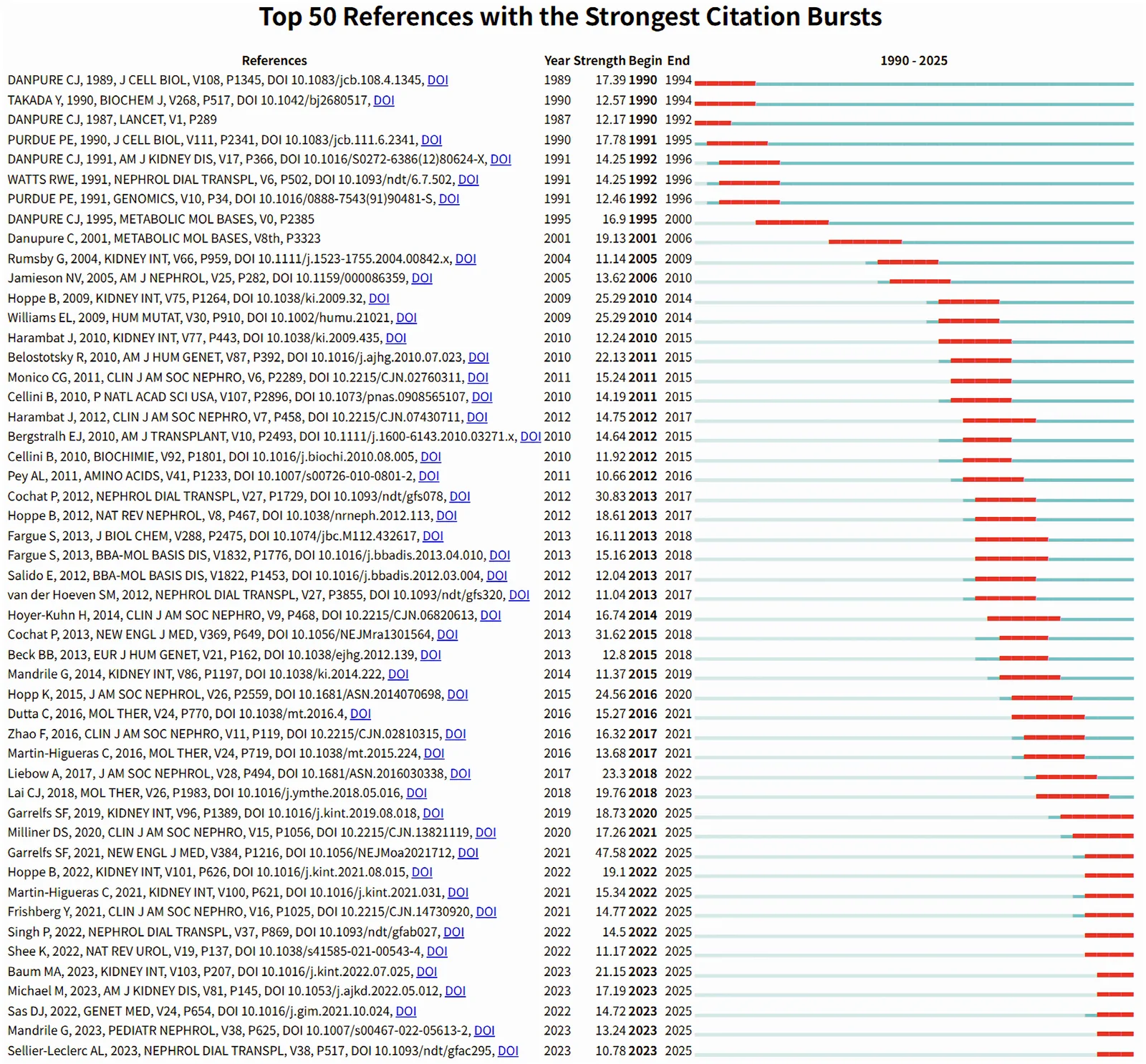
Top 50 references with the strongest citation bursts in primary hyperoxaluria research. Red bars indicate periods of strong citation bursts, reflecting references that attracted substantial attention during specific time intervals.

### 3.5. Keyword evolution and research hotspots

Keyword analysis revealed the progressive evolution of research themes. Early studies mainly focused on clinical manifestations, nephrocalcinosis, urolithiasis, and transplantation. Later studies increasingly emphasized genetic mutations, glyoxylate metabolism, and experimental models. Since 2020, RNA interference, glycolate oxidase, lactate dehydrogenase, and AGXT mutations have become major research hotspots (Table 7, Fig. 8, and Table S4, Supplemental Digital Content 4). This indicates a shift from symptomatic and surgical management to molecularly targeted therapeutic strategies.

**Table 7:** Top 20 high-frequency keywords in primary hyperoxaluria research.

Rank	Keyword	Counts	Rank	Keyword	Counts
1	Primary hyperoxaluria	469	11	Urolithiasis	84
2	Oxalate	194	12	Alanine	75
3	Children	155	13	glyoxylate aminotransferase	72
4	Diagnosis	139	14	Pyridoxine	71
5	Alanine glyoxylate aminotransferase	135	15	Urinary oxalate	67
6	Oxalosis	129	16	Liver transplantation	60
7	Disease	106	17	Kidney transplantation	55
8	Nephrocalcinosis	96	18	Phenotype	51
9	Transplantation	88	19	Primary oxalosis	51
10	Mutations	87	20	Dialysis	50

**Figure 8.**
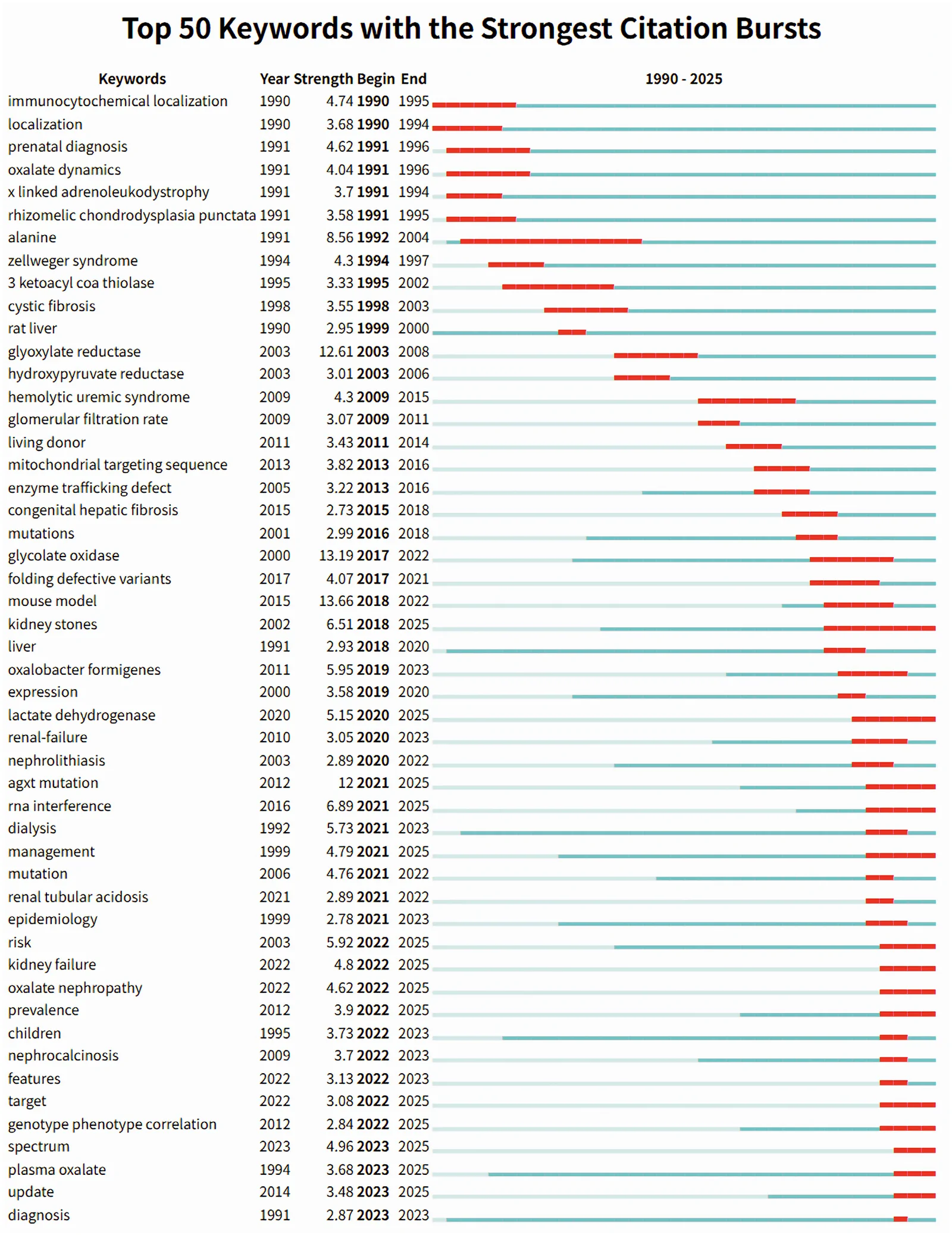
Top 50 keywords with the strongest citation bursts in primary hyperoxaluria research. Red bars indicate burst periods, reflecting emerging or rapidly increasing research topics during specific time intervals.

### 3.6. Keyword clustering and intellectual structure

Keyword clustering identified several major thematic areas. The first cluster focused on clinical characterization and diagnosis, including nephrocalcinosis, urolithiasis, and phenotype–genotype correlations. The second cluster reflected transplantation-based management, especially combined liver–kidney transplantation. The third cluster was centered on the molecular mechanisms, including AGXT mutations and glyoxylate metabolism. The most recent cluster focused on RNA interference and targeted metabolic therapies (Fig. 9A, B).

**Figure 9.**
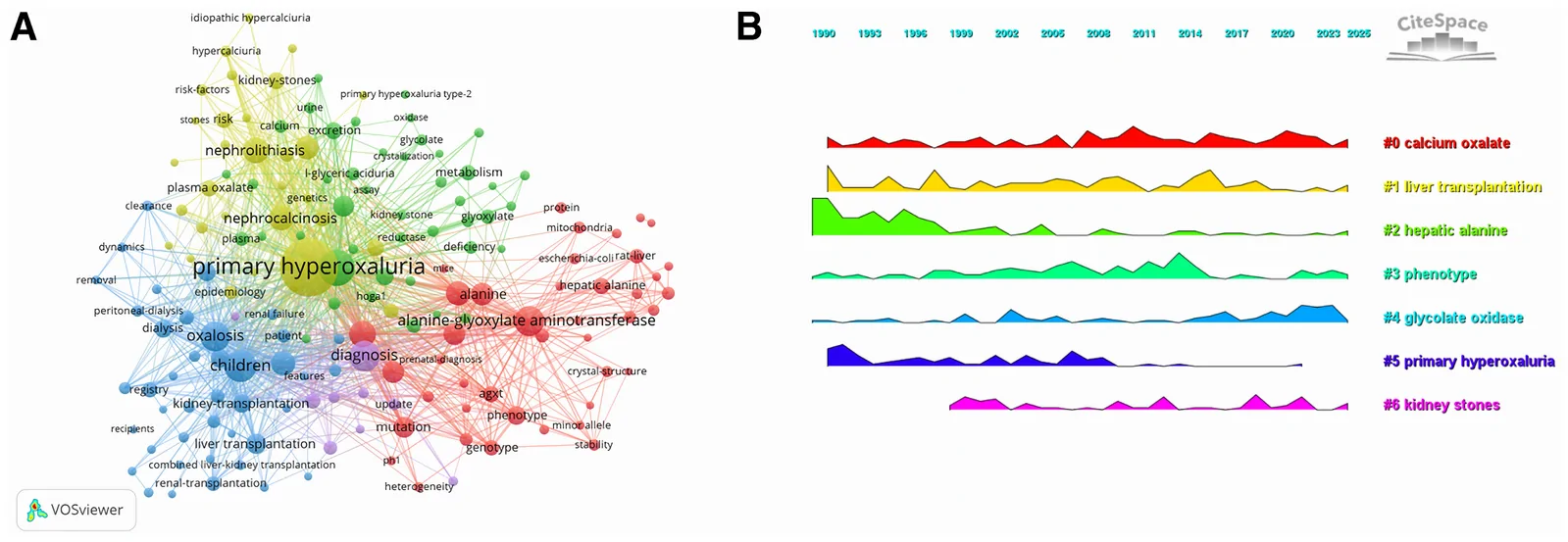
Keyword co-occurrence and clustering analysis in primary hyperoxaluria research. (A) Keyword co-occurrence network. (B) Keyword clustering map. Node size represents keyword frequency, and links indicate co-occurrence relationships.

Overall, the intellectual structure of the field has evolved from clinical observation to molecular mechanism exploration, and more recently, to precision therapeutic intervention.

## 4. Discussion

### 4.1. Principal findings

This bibliometric study systematically maps the global research landscape of PH from 1990 to 2025. PH research has evolved from clinical characterization and supportive management to investigations of molecular mechanisms and ultimately to precision therapeutic strategies. Early molecular studies helped to establish the genetic basis of PH, providing the foundation for later genotype-based diagnosis and mechanistic research.^[16]^ The subsequent expansion of next-generation sequencing further improved the identification of rare inherited disorders and likely contributed to the growth of PH-related publications after 2008.^[17]^

### 4.2. Evolution of research focus

The early phase of PH research mainly focused on the clinical manifestations, diagnosis, and management of renal complications. With advances in molecular diagnosis, the research focus has gradually shifted toward pathogenic genes, metabolic pathways, and enzyme dysfunction. This transition reflects a broader movement in rare disease research from phenotype-based classification to genotype-informed understanding and individualized management.^[16,17]^

The marked increase in publications after 2020 may be closely related to the clinical translation of RNA interference-based therapies. Lumasiran, the first approved RNA interference therapeutic for PH1, has attracted substantial academic attention and likely served as an important turning point in the field.^[18]^

### 4.3. RNA interference as a current research frontier

RNA interference has become a central research topic in recent years. Unlike conventional supportive treatment or organ replacement, RNA interference-based therapies directly target the hepatic metabolic pathways involved in oxalate production. Nedosiran, which targets lactate dehydrogenase, has also been evaluated as an RNA interference-based therapeutic strategy for PH and has shown pharmacodynamic activity in early clinical investigations.^[19]^

Long-term data support the sustained biochemical efficacy of RNA interference therapy. Clinical evidence has demonstrated persistent urinary oxalate reduction and suggested potential renoprotective effects over extended treatment periods. In addition, follow-up data from patients with advanced PH1 and severe renal impairment indicate that lumasiran may remain effective and generally safe in this high-risk population.^[20]^ However, despite these encouraging findings, long-term real-world outcomes, durability of response, comparative effectiveness, and cost-effectiveness require further investigation.^[21]^

### 4.4. Emerging role of gene-editing technologies

Gene-editing technologies, including clustered regularly interspaced short palindromic repeats (CRISPR)/CRISPR-associated protein 9 and base editing, represent emerging areas of interest in PH research. The successful clinical translation of CRISPR-based therapies to other monogenic diseases has encouraged the exploration of similar strategies for inherited metabolic disorders.^[22–24]^ In PH, preclinical studies have demonstrated the feasibility of targeting enzymes involved in oxalate metabolism, including glycolate oxidase and lactate dehydrogenase, as potential strategies to reduce oxalate production.^[25–29]^

Recent experimental studies have suggested that gene-editing strategies may provide a potential route toward durable metabolic correction in PH.^[30]^ Nevertheless, these technologies remain at an early stage, and additional translational studies are required. Future studies should focus on long-term safety, off-target effects, durability, ethical considerations, and the balance between one-time genetic interventions and chronic RNA interference therapy.

### 4.5. Global collaboration and research imbalance

The dominance of the United States and European countries is likely supported by well-established rare disease networks, multicenter collaborations, and sustained investment in orphan disease research, which have facilitated high-quality clinical studies and therapeutic innovation.^[31,32]^ Although China has shown increasing research activity, further international collaboration may enhance its global scientific impact.

Our findings also highlight several research gaps. Most evidence is derived from Europe and North America, whereas data from other regions remain limited. In addition, although RNA interference therapies have demonstrated promising efficacy, long-term real-world evidence is still accumulating,^[20,21]^ while the clinical translation of gene-editing strategies remains at an early stage.^[30]^ Addressing these gaps will be important for advancing precision medicine in PH.

### 4.6. Limitations

This study has several limitations. First, although the search strategy was supplemented with subtype-, gene-, and therapy-specific terms and manually verified to minimize omissions, the analysis was limited to the Web of Science Core Collection and English-language publications. Relevant studies indexed only in other databases, such as Scopus or Embase, or published in non-English journals may therefore have been missed. Second, although institutional names were standardized using a detailed thesaurus and manual verification, variations in affiliations, institutional aliases, and historical name changes may still have resulted in minor attribution errors. Third, because the data were collected only through September 15, 2025, the 2025 publication count represents partial-year output and may underestimate the full annual total; longitudinal trends involving 2025 should therefore be interpreted with caution. The inclusion of partial-year data for 2025 may affect the fitted trend. In addition, citation-based indicators may underestimate the influence of recently published studies because of citation lag. Finally, bibliometric analysis primarily characterizes publication patterns, collaboration networks, intellectual structures, and thematic evolution but cannot directly evaluate the novelty, disruptive influence, clinical efficacy, or policy translation of individual studies. Accordingly, therapeutic trends should be interpreted alongside evidence from clinical trials, extension studies, and real-world cohorts.

## Acknowledgments

The authors thank all researchers whose work contributed to this study.

## Author contributions

**Conceptualization:** Xiao Han, Shaozhong Xian, Yukun Liu.

**Data curation:** Xiao Han, Shaozhong Xian, Yukun Liu.

**Formal analysis:** Xiao Han, Shaozhong Xian, Yukun Liu.

**Investigation:** Xiao Han, Shaozhong Xian, Yukun Liu.

**Methodology:** Xiao Han, Shaozhong Xian, Yukun Liu.

**Project administration:** Xiao Han, Shaozhong Xian, Yukun Liu.

**Resources:** Xiao Han, Shaozhong Xian, Yukun Liu.

**Software:** Xiao Han, Shaozhong Xian, Yukun Liu.

**Supervision:** Xiao Han, Shaozhong Xian, Yukun Liu.

**Validation:** Xiao Han, Shaozhong Xian, Yukun Liu.

**Visualization:** Xiao Han, Shaozhong Xian, Yukun Liu.

**Writing – original draft:** Xiao Han, Yukun Liu.

**Writing – review & editing:** Xiao Han, Yukun Liu.








