# Supplementary material for: From symptomatic management to precision medicine: A bibliometric analysis and visualization of global research trends in primary hyperoxaluria (1990–2025)
Source: Medicine (Baltimore). 2026 Oct 23;105(43):e51250. doi: 10.1097/MD.0000000000051250 (PMC13706405; doi:10.1097/MD.0000000000051250)
Supplement: Supplementary file 1 [file medi-105-e51250-s001.docx]

| **Supplementary Table S1. Country/region collaboration metrics in primary hyperoxaluria research.** | | | | |
| --- | --- | --- | --- | --- |
| **ID** | **Country/Region** | **Documents** | **Citations** | **Total Link Strength** |
| 78 | United States | 542 | 15188 | 455 |
| 19 | England | 222 | 7201 | 407 |
| 22 | Germany | 213 | 5988 | 381 |
| 21 | France | 176 | 4962 | 388 |
| 31 | Italy | 162 | 3488 | 197 |
| 64 | Spain | 143 | 2640 | 225 |
| 45 | Netherlands | 118 | 3440 | 357 |
| 49 | China | 99 | 936 | 25 |
| 30 | Israel | 67 | 1726 | 179 |
| 7 | Belgium | 60 | 1488 | 160 |
| 32 | Japan | 53 | 860 | 41 |
| 10 | Canada | 45 | 1249 | 65 |
| 26 | India | 43 | 310 | 26 |
| 68 | Switzerland | 40 | 1511 | 112 |
| 73 | Turkey | 35 | 192 | 21 |
| 3 | Australia | 26 | 711 | 97 |
| 72 | Tunisia | 24 | 286 | 21 |
| 18 | Egypt | 20 | 553 | 42 |
| 51 | Poland | 15 | 278 | 43 |
| 36 | Lebanon | 14 | 287 | 84 |
| 56 | Saudi Arabia | 14 | 209 | 14 |
| 75 | United Arab Emirates | 14 | 485 | 102 |
| 48 | Pakistan | 13 | 210 | 32 |
| 27 | Iran | 12 | 145 | 3 |
| 55 | Russia | 12 | 61 | 42 |
| 62 | South Africa | 12 | 332 | 27 |
| 67 | Sweden | 12 | 266 | 36 |
| 8 | Brazil | 11 | 250 | 21 |
| 23 | Greece | 11 | 53 | 31 |
| 4 | Austria | 10 | 348 | 28 |
| 33 | Jordan | 10 | 158 | 70 |
| 46 | New Zealand | 10 | 347 | 6 |
| 47 | Norway | 8 | 96 | 3 |
| 63 | South Korea | 8 | 159 | 10 |
| 43 | Morocco | 7 | 73 | 5 |
| 40 | Macedonia | 6 | 245 | 15 |
| 52 | Portugal | 6 | 151 | 5 |
| 54 | Romania | 6 | 74 | 20 |
| 69 | Syria | 5 | 10 | 1 |
| 70 | Taiwan | 5 | 102 | 8 |
