# Supplementary material for: From symptomatic management to precision medicine: A bibliometric analysis and visualization of global research trends in primary hyperoxaluria (1990–2025)
Source: Medicine (Baltimore). 2026 Oct 23;105(43):e51250. doi: 10.1097/MD.0000000000051250 (PMC13706405; doi:10.1097/MD.0000000000051250)
Supplement: Supplementary file 2 [file medi-105-e51250-s002.docx]

| **Supplementary Table S2. Journal publication and citation metrics in primary hyperoxaluria research.** | | | | |
| --- | --- | --- | --- | --- |
| **ID** | **Journal** | **Documents** | **Citations** | **Total Link Strength** |
| 406 | *Pediatric Nephrology* | 171 | 2045 | 1143 |
| 378 | *Nephrology Dialysis Transplantation* | 77 | 1609 | 803 |
| 321 | *Journal of the American Society of Nephrology* | 66 | 1215 | 395 |
| 329 | *Kidney International* | 48 | 2978 | 1121 |
| 409 | *Pediatric Transplantation* | 44 | 437 | 317 |
| 22 | *American Journal of Kidney Diseases* | 40 | 947 | 345 |
| 324 | *Journal of Urology* | 29 | 410 | 108 |
| 470 | *Transplantation* | 28 | 537 | 236 |
| 32 | *American Journal of Transplantation* | 27 | 536 | 246 |
| 483 | *Urolithiasis* | 25 | 554 | 363 |
| 223 | *Human Gene Therapy* | 24 | 9 | 5 |
| 362 | *Molecular therapy* | 24 | 292 | 106 |
| 330 | *Kidney International Reports* | 20 | 224 | 392 |
| 336 | *Liver Transplantation* | 19 | 365 | 119 |
| 76 | *Biochimica et biophysica acta-molecular basis of disease* | 15 | 597 | 321 |
| 268 | *Journal of Biological Chemistry* | 14 | 828 | 183 |
| 293 | *Journal of Inherited Metabolic Disease* | 14 | 184 | 93 |
| 70 | *Biochemical Journal* | 13 | 504 | 162 |
| 96 | *BMC Nephrology* | 13 | 121 | 150 |
| 128 | *Clinical Kidney Journal* | 13 | 135 | 218 |
| 361 | *Molecular Genetics and Metabolism* | 13 | 259 | 204 |
| 215 | *Hepatology* | 12 | 215 | 15 |
| 25 | *American Journal of Nephrology* | 11 | 206 | 127 |
| 486 | *Urological Research* | 11 | 120 | 39 |
| 306 | *Journal of Nephrology* | 10 | 66 | 110 |
| 469 | *Transplant International* | 10 | 88 | 22 |
| 127 | *Clinical Journal of the American Society of Nephrology* | 9 | 707 | 359 |
| 141 | *Comptes rendus chimie* | 9 | 210 | 36 |
| 199 | *Frontiers in Pediatrics* | 9 | 67 | 140 |
| 44 | *Annals of Clinical Biochemistry* | 8 | 221 | 130 |
| 79 | *Biochimie* | 8 | 187 | 102 |
| 145 | *Cureus Journal of Medical Science* | 8 | 10 | 41 |
| 485 | *Urologic Clinics of North America* | 8 | 219 | 83 |
| 115 | *Clinica Chimica Acta* | 7 | 132 | 33 |
| 129 | *Clinical Nephrology* | 7 | 39 | 24 |
| 151 | *Current Opinion in Nephrology and Hypertension* | 7 | 202 | 139 |
| 374 | *Nature Reviews Nephrology* | 7 | 644 | 329 |
| 377 | *Nephrology* | 7 | 18 | 46 |
| 379 | *Nephron* | 7 | 144 | 52 |
| 444 | *Scientific Reports* | 7 | 48 | 77 |
| 78 | *Biochimica et Biophysica Acta-proteins and Proteomics* | 6 | 180 | 151 |
| 123 | *Clinical Chemistry* | 6 | 167 | 67 |
| 177 | *European journal of pediatrics* | 6 | 170 | 86 |
| 225 | *Human Molecular Genetics* | 6 | 313 | 120 |
| 226 | *Human Mutation* | 6 | 279 | 199 |
| 407 | *Pediatric Radiology* | 6 | 43 | 29 |
| 418 | *Plos one* | 6 | 199 | 124 |
| 423 | *Proceedings of the National Academy of Sciences of the United States of America* | 6 | 527 | 172 |
| 457 | *Swiss Medical Weekly* | 6 | 0 | 0 |
| 472 | *Transplantation Proceedings* | 6 | 49 | 16 |
