# Supplementary material for: From symptomatic management to precision medicine: A bibliometric analysis and visualization of global research trends in primary hyperoxaluria (1990–2025)
Source: Medicine (Baltimore). 2026 Oct 23;105(43):e51250. doi: 10.1097/MD.0000000000051250 (PMC13706405; doi:10.1097/MD.0000000000051250)
Supplement: Supplementary file 3 [file medi-105-e51250-s003.docx]

| **Supplementary Table S3. Author publication and citation metrics in primary hyperoxaluria research.** | | | | |
| --- | --- | --- | --- | --- |
| **ID** | **Author** | **Documents** | **Citations** | **Total Link Strength** |
| 2341 | Hoppe, Bernd | 90 | 2457 | 126 |
| 1061 | Cochat, Pierre | 45 | 2399 | 109 |
| 1208 | Danpure,CJ | 42 | 2379 | 5 |
| 3180 | Lieske, John c. | 66 | 2060 | 135 |
| 3663 | Milliner, Dawn s. | 47 | 1997 | 70 |
| 4649 | Rumsby, Gill | 31 | 1777 | 49 |
| 4697 | Salido, Eduardo | 51 | 1442 | 41 |
| 1645 | Fargue, Sonia | 26 | 1239 | 35 |
| 2057 | Groothoff, Jaap w. | 37 | 1150 | 101 |
| 1785 | Frishberg, Yaacov | 44 | 1127 | 134 |
| 467 | Beck, Bodo B. | 29 | 1048 | 67 |
| 895 | Cellini, Barbara | 45 | 1013 | 40 |
| 2424 | Hulton, Sally-Anne | 24 | 904 | 90 |
| 3378 | Magen, Daniella | 28 | 824 | 118 |
| 4754 | Sas, David j. | 21 | 757 | 65 |
| 3449 | Marangella, m | 27 | 756 | 24 |
| 1870 | Garrelfs, Sander f. | 27 | 728 | 91 |
| 4252 | Petrarulo, m | 24 | 710 | 24 |
| 2853 | Knight, John | 29 | 648 | 27 |
| 4066 | Oppici, Elisa | 22 | 648 | 27 |
| 3435 | Mandrile, Giorgia | 20 | 608 | 49 |
| 4953 | Shasha-lavsky, Hadas | 22 | 551 | 98 |
| 356 | Bacchetta, Justine | 45 | 541 | 70 |
| 3488 | Martin-higueras, Cristina | 25 | 365 | 36 |
| 4882 | Sellier-leclerc, Anne-laure | 28 | 246 | 89 |
