# Supplementary material for: From symptomatic management to precision medicine: A bibliometric analysis and visualization of global research trends in primary hyperoxaluria (1990–2025)
Source: Medicine (Baltimore). 2026 Oct 23;105(43):e51250. doi: 10.1097/MD.0000000000051250 (PMC13706405; doi:10.1097/MD.0000000000051250)
Supplement: Supplementary file 4 [file medi-105-e51250-s004.docx]

| **Supplementary Table S4. Keyword co-occurrence metrics in primary hyperoxaluria research.** | | | |
| --- | --- | --- | --- |
| **ID** | **Keyword** | **Occurrences** | **Total Link Strength** |
| 2898 | Primary Hyperoxaluria | 469 | 1900 |
| 2575 | Oxalate | 194 | 998 |
| 601 | Children | 155 | 809 |
| 962 | Diagnosis | 139 | 754 |
| 162 | Alanine-Glyoxylate Aminotransferase | 135 | 662 |
| 2626 | Oxalosis | 129 | 628 |
| 1000 | Disease | 106 | 524 |
| 2448 | Nephrocalcinosis | 96 | 515 |
| 3651 | Transplantation | 88 | 473 |
| 2409 | Mutations | 87 | 411 |
| 3772 | Urolithiasis | 84 | 467 |
| 153 | Alanine | 75 | 397 |
| 1453 | Glyoxylate Aminotransferase | 72 | 386 |
| 3049 | Pyridoxine | 71 | 452 |
| 3748 | Urinary Oxalate | 67 | 357 |
| 2105 | Liver Transplantation | 60 | 265 |
| 1984 | Kidney Transplantation | 55 | 319 |
| 2777 | Phenotype | 51 | 292 |
| 2933 | Primary Oxalosis | 51 | 269 |
| 968 | Dialysis | 50 | 290 |
| 2385 | Mouse Model | 49 | 251 |
| 1234 | Failure | 47 | 256 |
| 1533 | Hemodialysis | 47 | 283 |
| 1970 | Kidney Stones | 46 | 255 |
| 1206 | Excretion | 44 | 241 |
| 3210 | Renal Failure | 43 | 196 |
| 465 | Calcium Oxalate | 42 | 215 |
| 2820 | Plasma Oxalate | 41 | 210 |
| 2252 | Metabolism | 38 | 162 |
| 1392 | Genotype | 36 | 219 |
| 3151 | Registry | 35 | 177 |
| 2185 | Management | 34 | 175 |
| 1452 | Glyoxylate | 32 | 140 |
| 2089 | Liver | 32 | 149 |
| 3584 | Therapy | 32 | 161 |
| 2623 | Oxalobacter-Formigenes | 30 | 166 |
| 2673 | Patient | 30 | 99 |
| 2153 | Lumasiran | 29 | 122 |
| 2723 | Peroxisomal Alanine | 29 | 170 |
| 2816 | Plasma | 29 | 158 |
| 460 | Calcium | 27 | 133 |
| 911 | Deficiency | 26 | 105 |
| 3490 | Subcellular-Distribution | 26 | 76 |
| 2010 | L-Glyceric Aciduria | 25 | 124 |
| 38 | 4-Hydroxy-2-Oxoglutarate Aldolase | 23 | 148 |
| 2826 | Point Mutation | 23 | 105 |
| 3715 | Update | 23 | 115 |
| 2311 | Mitochondria | 22 | 102 |
| 72 | Acid | 21 | 83 |
| 639 | Chronic Kidney Disease | 21 | 112 |
| 1394 | Genotype-Phenotype Correlation | 21 | 134 |
| 1701 | Hypercalciuria | 21 | 95 |
| 2405 | Mutation Analysis | 21 | 121 |
| 2889 | Prevalence | 21 | 91 |
| 2911 | Primary Hyperoxaluria Type 2 | 21 | 80 |
| 3127 | Recurrence | 21 | 99 |
| 3257 | Risk-Factors | 21 | 84 |
| 3765 | Urine | 21 | 95 |
| 841 | Crystal-Structure | 20 | 82 |
| 1171 | Escherichia-Coli | 20 | 81 |
| 1215 | Expression | 20 | 85 |
| 1438 | Glycolate | 20 | 120 |
| 1626 | HOGA1 | 20 | 104 |
| 2717 | Peritoneal-Dialysis | 20 | 149 |
| 2969 | Protein | 20 | 72 |
| 3054 | Pyruvate Aminotransferase | 20 | 98 |
| 3443 | Stability | 20 | 101 |
| 2616 | Oxalic-Acid | 19 | 116 |
| 3262 | RNA Interference | 19 | 91 |
| 720 | Combined Liver-Kidney Transplantation | 18 | 120 |
| 880 | Cystinuria | 18 | 89 |
| 2330 | Model | 18 | 65 |
| 3134 | Reductase | 18 | 88 |
| 663 | Clearance | 17 | 114 |
| 1483 | GRHPR | 17 | 108 |
| 3334 | Serine | 17 | 88 |
| 3520 | Survival | 17 | 70 |
| 1061 | Dynamics | 16 | 105 |
| 1385 | Genetics | 16 | 98 |
| 1538 | Hemolytic-Uremic Syndrome | 16 | 48 |
| 1599 | Heterogeneity | 16 | 92 |
| 1690 | Hydroxyproline | 16 | 60 |
| 2750 | Peroxisomes | 16 | 58 |
| 2915 | Primary Hyperoxaluria Type I | 16 | 73 |
| 3030 | Purification | 16 | 70 |
| 571 | Cells | 15 | 47 |
| 1157 | Epithelial-Cells | 15 | 59 |
| 1463 | Glyoxylate Reductase | 15 | 73 |
| 3425 | Spectrum | 15 | 67 |
| 3657 | Treatment | 15 | 92 |
| 292 | Assay | 14 | 69 |
| 655 | Citrate | 14 | 81 |
| 670 | Clinical Heterogeneity | 14 | 93 |
| 1439 | Glycolate Excretion | 14 | 86 |
| 1485 | Growth | 14 | 55 |
| 1748 | Idiopathic Hypercalciuria | 14 | 43 |
| 2589 | Oxalate Dynamics | 14 | 87 |
| 3161 | Removal | 14 | 93 |
| 3537 | Systemic Oxalosis | 14 | 89 |
| 974 | Diet | 13 | 79 |
| 1259 | Features | 13 | 61 |
| 1520 | Healthy-Subjects | 13 | 80 |
| 1829 | Inflammation | 13 | 58 |
| 2304 | Minor Allele | 13 | 80 |
| 2312 | Mitochondrial | 13 | 68 |
| 3204 | Renal Tubular-Acidosis | 13 | 44 |
| 3246 | Rhizomelic Chondrodysplasia Punctata | 13 | 17 |
| 3749 | Urinary Oxalate Excretion | 13 | 65 |
| 3789 | Variants | 13 | 69 |
| 187 | Allograft Survival | 12 | 89 |
| 875 | Cystic-Fibrosis | 12 | 34 |
| 1129 | Enteric Hyperoxaluria | 12 | 55 |
| 1634 | Homozygous Familial Hypercholesterolemia | 12 | 19 |
| 1770 | Immunocytochemical Localization | 12 | 54 |
| 1794 | Inborn Errors of Metabolism | 12 | 53 |
| 2267 | Methylmalonic Acidemia | 12 | 62 |
| 2275 | Mice | 12 | 41 |
| 2439 | Nedosiran | 12 | 53 |
| 2628 | Oxidase | 12 | 64 |
| 2884 | Prenatal-Diagnosis | 12 | 47 |
| 3281 | Saccharomyces-Cerevisiae | 12 | 27 |
| 3372 | Single-Center Experience | 12 | 64 |
| 216 | Aminotransferase | 11 | 73 |
| 848 | Crystallization | 11 | 51 |
| 945 | Deposition | 11 | 55 |
| 993 | Dimerization | 11 | 51 |
| 1145 | Enzyme Trafficking Defect | 11 | 92 |
| 1696 | Hydroxypyruvate Reductase | 11 | 53 |
| 1821 | Infants | 11 | 58 |
| 2120 | Living Donor | 11 | 51 |
| 2325 | Mitochondrial Targeting Sequence | 11 | 68 |
| 2600 | Oxalate Nephropathy | 11 | 33 |
| 2697 | Pediatrics | 11 | 52 |
| 2732 | Peroxisomal L-Alanine | 11 | 66 |
| 3090 | Rat | 11 | 63 |
| 3099 | Rat-Liver Peroxism | 11 | 17 |
| 3119 | Recipients | 11 | 30 |
| 3135 | Reductase GRHPR | 11 | 76 |
| 3211 | Renal-Function | 11 | 60 |
| 3353 | Shock-Wave Lithotripsy | 11 | 53 |
| 3478 | Stones | 11 | 55 |
| 3479 | Strategy | 11 | 64 |
| 3652 | Transport | 11 | 58 |

Abbreviations: GRHPR, Glyoxylate reductase/hydroxypyruvate reductase; HOGA1, 4-hydroxy-2-oxoglutarate aldolase 1
